# Supplementary figures and images for: Early and transient increase in cortical pyramidal cell excitability and delayed alteration of evoked synaptic transmission and t-SNARE proteins content in the hippocampus and neocortex of neonatal and juvenile Stxbp1 heterozygous mice
Source: Front Cell Neurosci. 2026 May 18;20:1842951. doi: 10.3389/fncel.2026.1842951 (PMC13222804; doi:10.3389/fncel.2026.1842951)

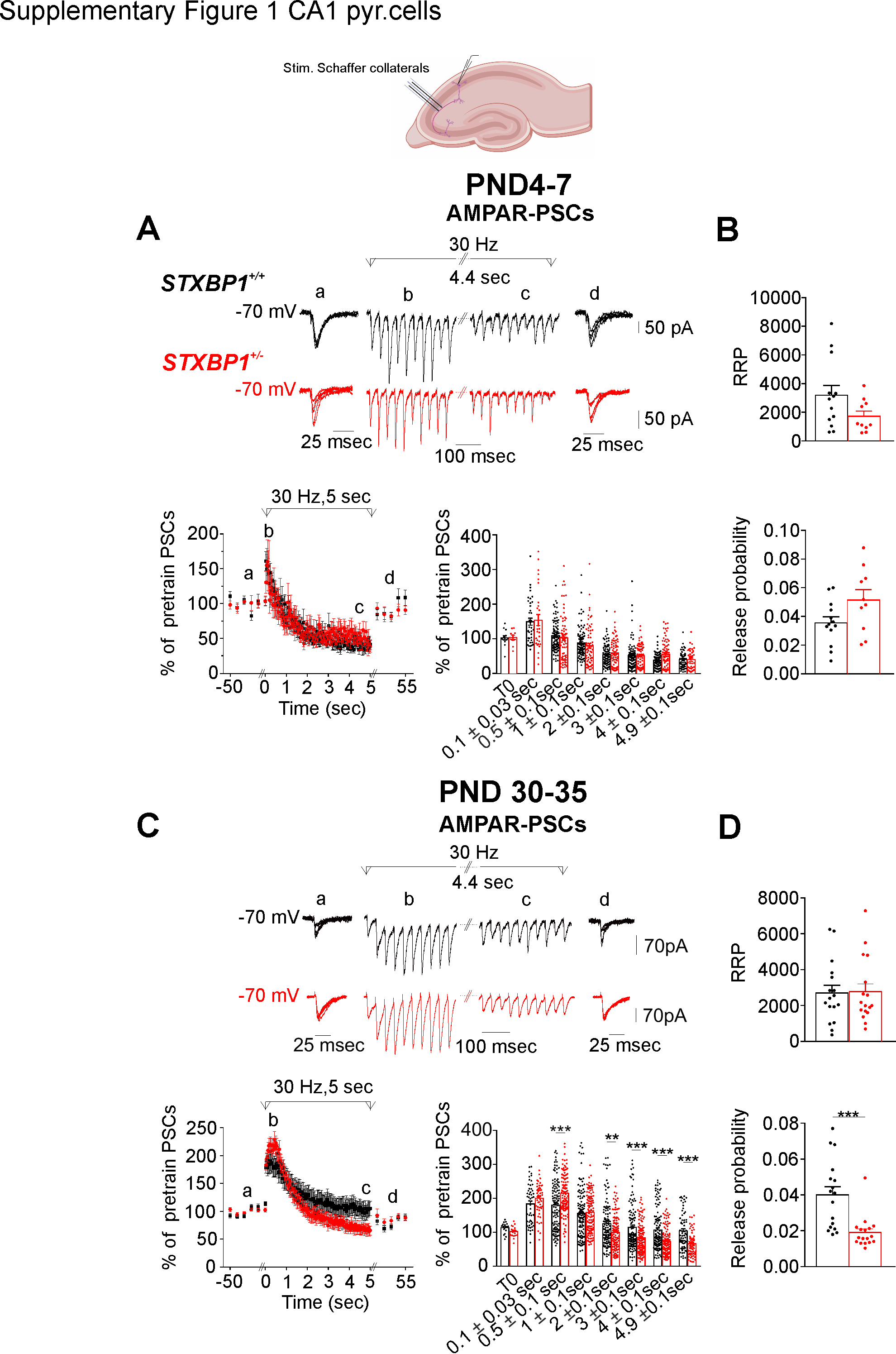

Supplement: SUPPLEMENTARY FIGURE 1 — Consequences of Munc18.1 deficiency on AMPA receptors mediated postsynaptic currents (AMPAR-PSCs) in CA1 pyramidal cells and evoked by electrical stimulation applied at 30 Hz in the stratum radiatum. Top: Schematic of the recorded region and experimental design (Biorender). (A) AMPAR mediated postsynaptic currents (AMPAR-PSCs) recorded in CA1 pyramidal cells from WT (black) and STXBP1+/− mice (red) aged 7 days old. Top: (a) 5 superimposed PSCs evoked at 0.1Hz before the stimulation at 30Hz; (b,c) PSCs during the first and the last second of the stimulation at 30 Hz stimulation (applied during 5 sec); (d) 5 superimposed PSCs evoked at 0.1Hz after the stimulation at 30Hz. Below on the left: Graph quantifying, at PND 4-7, the amplitude of PSCs (mean ± SEM), expressed as the percentage of 5 PSCs evoked at 0.1Hz before the stimulation at 30 Hz plus the first PSC of the train. Black: n = 13 cells / 3WT mice in which the stimulation protocol was applied 31 times; Red: n = 10 cells, /4 STXBP1+/− mice in which the stimulation protocol was applied 30 times. Right: Boxplots showing average values in each cells of relative AMPAR-PSCs during the stimulation at 30 Hz and grouped at times indicated in abscissa (B) RRP (upper boxplots) and probability of glutamate release (bottom boxplots) estimated at 30 Hz (Black: n = 13 cells / 3WT mice; red: n = 10 cells/4 STXBP1+/− mice). The size of the readily releasing pool of synaptic vesicles (RRP) was estimated after fitting a straight line to stimuli 100-150 and then back-extrapolating this line to the y-axis. (C) Same as (A), but for AMPAR-PSCs evoked by stimulation at 30 Hz during 5 sec in pyramidal cells from juvenile mice aged 30-35 days old. Black: n = 17 cells/ 4 WT mice in which the stimulation protocol was applied 45 times; Red: n = 17 cells / 4 STXBP1+/− mice in which the stimulation protocol was applied 47 times. Statistics at t = 0.5 ± 0.1 sec: Mann-Whitney’s test, p = 0.0002***, n = 119 WT AMPAR-PSCs and n = 119 STXBP1+/− ce [file Image_1.tif]

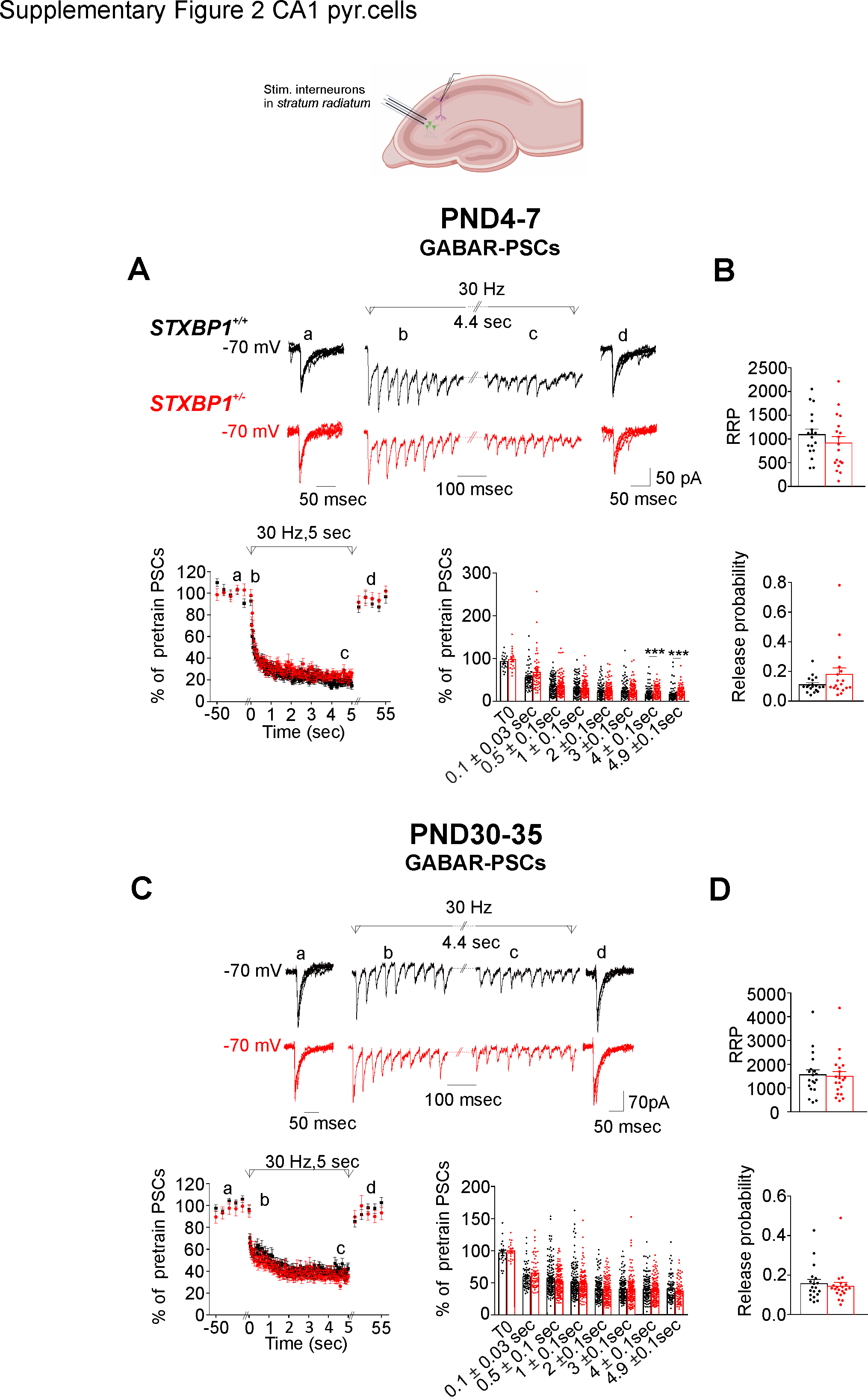

Supplement: SUPPLEMENTARY FIGURE 2 — Consequences of Munc18.1 deficiency on GABA receptors mediated postsynaptic currents (GABAR-PSCs) in CA1 pyramidal cells and evoked by electrical stimulation applied at 30 Hz in the stratum radiatum. Top: Schematic of the recorded region and experimental design (Biorender). (A) GABAR mediated postsynaptic currents (GABAR-PSCs) recorded in CA1 pyramidal cells from WT (black) and STXBP1+/− mice (red) aged 5 days old. (a) 5 superimposed PSCs evoked at 0.1Hz before the stimulation at 30Hz; (b,c) PSCs recorded during the first and the last second of the stimulation at 30 Hz stimulation; (d) 5 superimposed PSCs evoked at 0.1Hz after the stimulation at 30Hz. Below on the left: Graph quantifying, at PND 4-7, the amplitude of GABAR-PSCs (mean ± SEM, expressed as the percentage of 5 PSCs evoked at 0.1Hz before the stimulation at 30 Hz plus the first PSC of the train). Black: n = 18 cells / 3WT mice in which the stimulation protocol was applied 41 times; Red: n = 18 cells /3 STXBP1+/− mice in which the stimulation protocol was applied 53 times On the right: Boxplots showing average values in each cells of relative GABAR-PSCs during the stimulation at 30 Hz and grouped at times indicated in abscissa. Statistics at t = 4 ± 0.1 sec: Mann–Whitney’s test, p < 0.001*** n = 108 WT GABAR-PSCs and n = 126 STXBP1+/− GABAR-PSCs. Statistics at t = 4.9 ± 0.1 sec: Mann–Whitney’s test, p < 0.001*** n = 72 WT GABAR-PSCs and n = 72 STXBP1+/− GABAR-PSCs. (B) RRP (upper boxplots) and probability of GABA release (bottom boxplots) from Gabaergic inputs located in the stratum radiatum of CA1 from neonatal mice after stimulation at 30 Hz (n = 17 cells/3 WT mice and n = 18 cells/3 STXBP1+/− mice). (C) Same as (A), but GABAR-PSCs were recorded in pyramidal cells from juvenile mice aged 30-35 days old. a) Black: n = 21 cells/3 WT mice in which the stimulation protocol was applied 61 times. Red: n = 20 cells/ 3 STXBP1+/− mice in which the stimulation protocol was applied 59 times. (D) RRP and probabilit [file Image_2.tif]

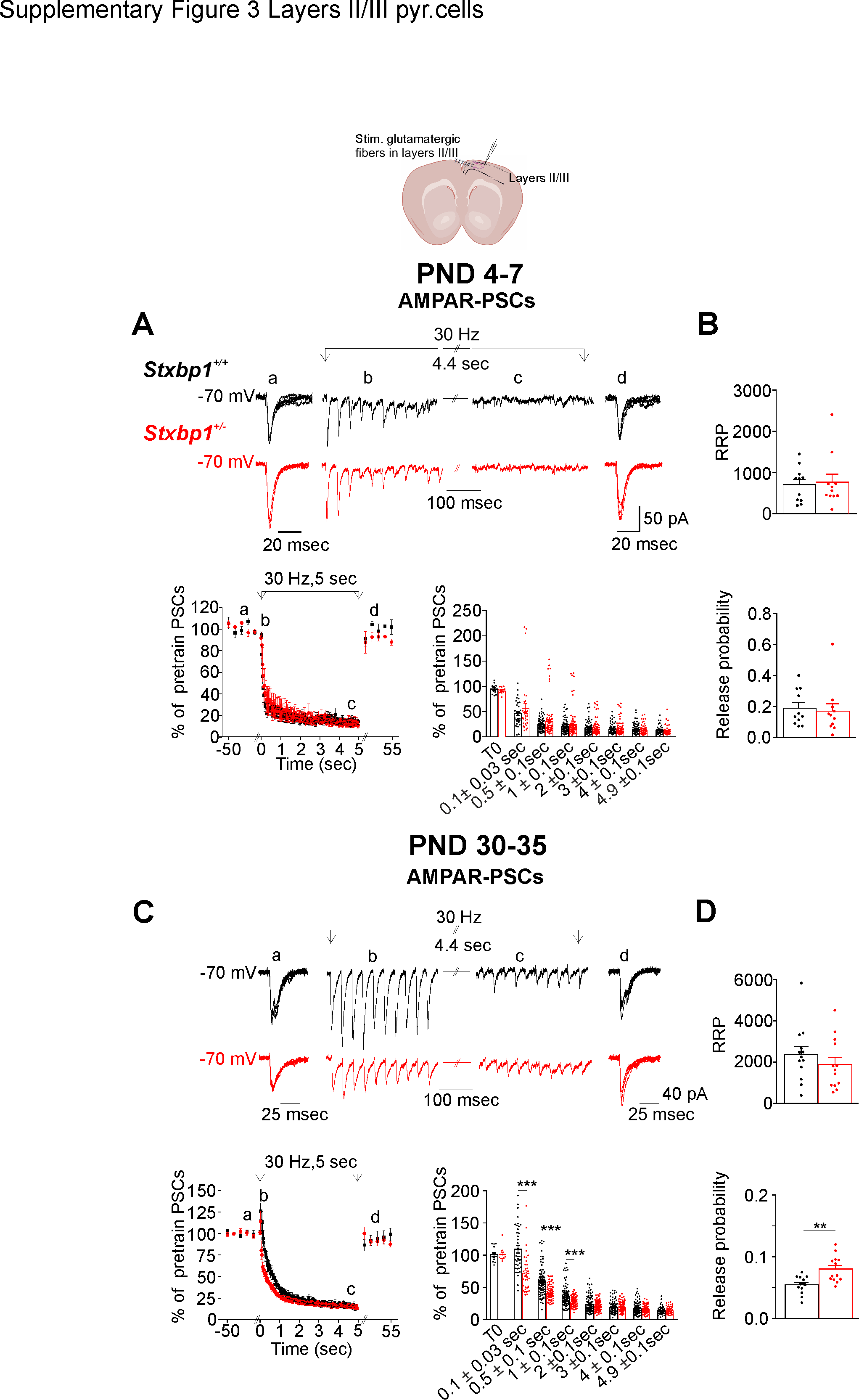

Supplement: SUPPLEMENTARY FIGURE 3 — Consequences of Munc18.1 deficiency on AMPAR- PSCs recorded in pyramidal cells of layers II/III of the motor cortex and evoked by electrical stimulation applied at 30 Hz in the layers II/III. Top: Schematic of the recorded region and experimental design (Biorender). (A) AMPAR mediated postsynaptic currents (AMPAR-PSCs) recorded in Layers II/III pyramidal cells from WT (black) and STXBP1+/- mice (red) aged 7 days old. Top: (a) 5 superimposed PSCs evoked at 0.1Hz before the stimulation at 30Hz; (b,c) PSCs recorded during the first and the last second of the stimulation at 30 Hz stimulation (applied during 5 sec); (d) 5 superimposed PSCs evoked at 0.1Hz after the stimulation at 30Hz. Below on the left: Graph quantifying, at PND 4–7, the amplitude of PSCs (mean ± SEM), expressed as the percentage of 5 PSCs evoked at 0.1Hz before the stimulation at 30 Hz plus the first PSC of the train. Black: n = 11 cells / 3WT mice in which the stimulation protocol was applied 21 times; red: n = 11 cells /3 STXBP1+/− mice in which the stimulation protocol was applied 30 times. On the right: Boxplots showing average values in each cells of relative AMPA-PSCs during the stimulation at 30 Hz and grouped at times indicated in abscissa. (B) RRP in layers II/III glutamatergic fibers in motor cortical slices and probability of glutamate release from neonatal WT and STXBP1+/− mice estimated at 30 Hz (Black: n = 11 cells / 3WT mice; red: n = 11 cells/3 STXBP1+/− mice). (C) Same as A but for AMPAR-PSCs evoked by stimulation at 30 Hz in pyramidal cells from juvenile mice aged 30-35 days old. Traces of from mice aged 30 days old. Black: n = 13 cells/ 3 WT mice in which the stimulation protocol was applied 30 times; Red: n = 13 cells / 3 STXBP1+/− mice in which the stimulation protocol was applied 35 times. Statistics at t = 0.1 ± 0.03 sec: Mann-Whitney’s test, p < 0.0001***, n = 37 WT AMPAR-PSCs and n = 37 STXBP1+/− AMPAR-PSCs. Statistics at t = 0.5 ± 0.1 sec: Mann–Whitney’s test, p < 0.0001***, n [file Image_3.tif]

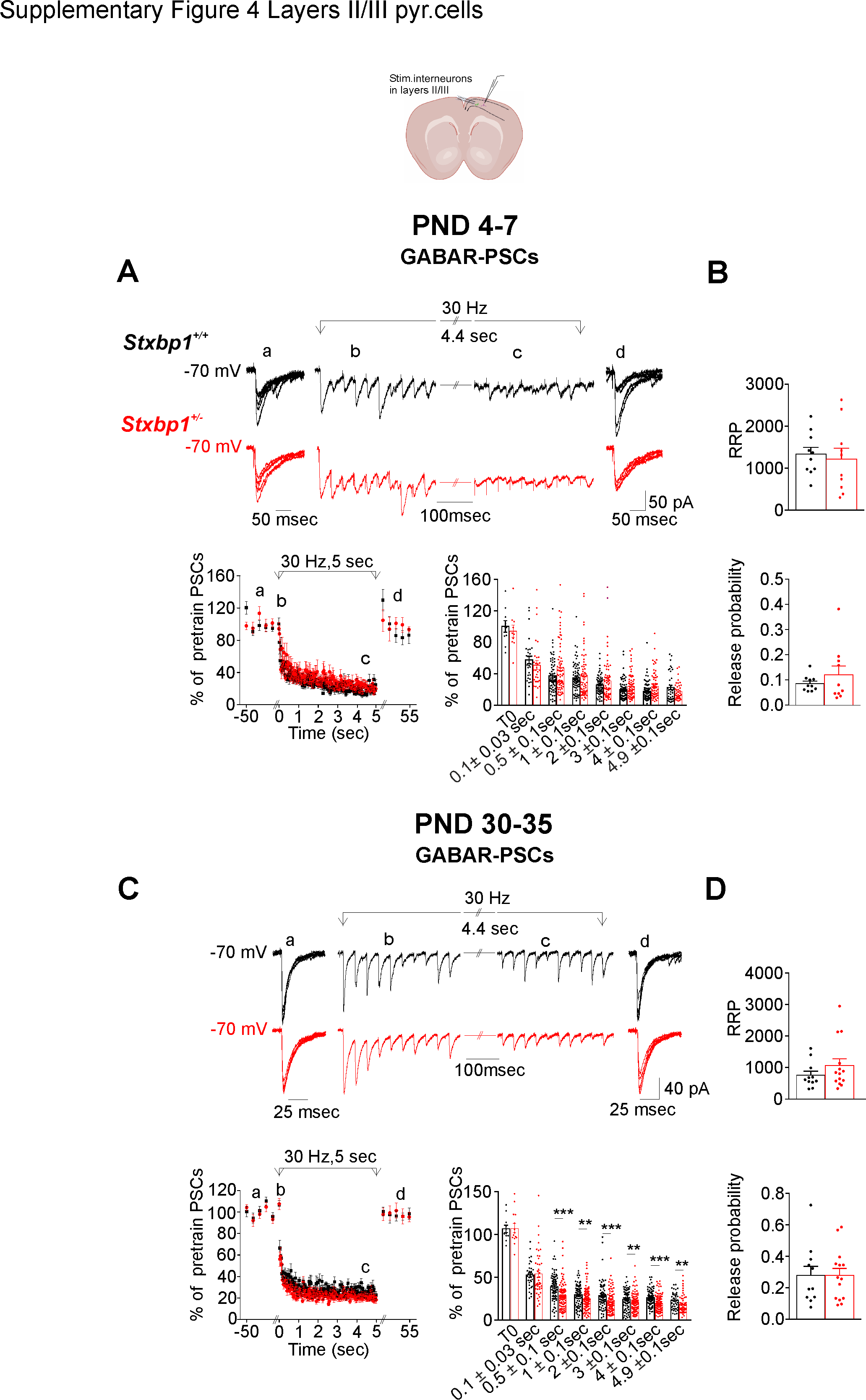

Supplement: SUPPLEMENTARY FIGURE 4 — Consequences of Munc18.1 deficiency on GABAR- PSCs recorded in pyramidal cells of layers II/III of the motor cortex and evoked by electrical stimulation applied at 30 Hz in the layers II/III. Top: Schematic of the recorded region and experimental design (Biorender). (A) GABAR mediated postsynaptic currents (GABAR-PSCs) recorded in pyramidal cells from WT (black) and STXBP1+/- mice (red) aged 7 days old. (a) 5 superimposed PSCs evoked at 0.1Hz before the stimulation at 30Hz; (b,c) PSCs recorded during the first and the last second of the stimulation at 10 Hz stimulation; (d) 5 superimposed PSCs evoked at 0.1Hz after the stimulation at 30Hz. Below on the left: Graph quantifying, at PND 4-7, the amplitude of GABAR-PSCs (mean ± SEM), expressed as the percentage of 5 PSCs evoked at 0.1Hz before the stimulation at 30 Hz plus the first PSC of the train. Black: n = 10 cells / 3WT mice in which the stimulation protocol was applied 21 times; red: n = 10 cells, /3 STXBP1+/− mice in which the stimulation protocol was applied 30 times. (B) RRP in layers II/III Gabaergic fibers in motor cortical slices and probability of GABA release from neonatal WT and STXBP1+/− mice estimated at 30 Hz: n = 10 cells / 3WT mice and n = 10 cells/3 STXBP1+/− mice. (C) Same as (A), but for GABAR-PSCs evoked by stimulation at 30 Hz and recorded in pyramidal cells from juvenile mice aged 30–35 days old. Black: n = 11 cells / 3WT mice in which the stimulation protocol was applied 25 times; red: n = 14 cells, /3 STXBP1+/− mice in which the stimulation protocol was applied 38 times. Statistics at t = 0.5 ± 0.1 sec: Mann–Whitney’s test, p < 0.0001***, n = 77 WT GABAR-PSCs and n = 98 STXBP1+/− GABAR-PSCs. Statistics at t = 1 ± 0.1 sec: Mann-Whitney’s test, p = 0.0065**, n = 77 WT GABAR-PSCs and n = 98 STXBP1+/− GABAR-PSCs. Statistics at t = 2 ± 0.1 sec: Mann–Whitney’s test, p = 0.0002***, n = 77 WT GABAR-PSCs, and n = 98 STXBP1+/− GABAR-PSCs. Statistics at t = 3 ± 0.1 sec: Mann–Whitney’s test, p = 0.0071* [file Image_4.tif]

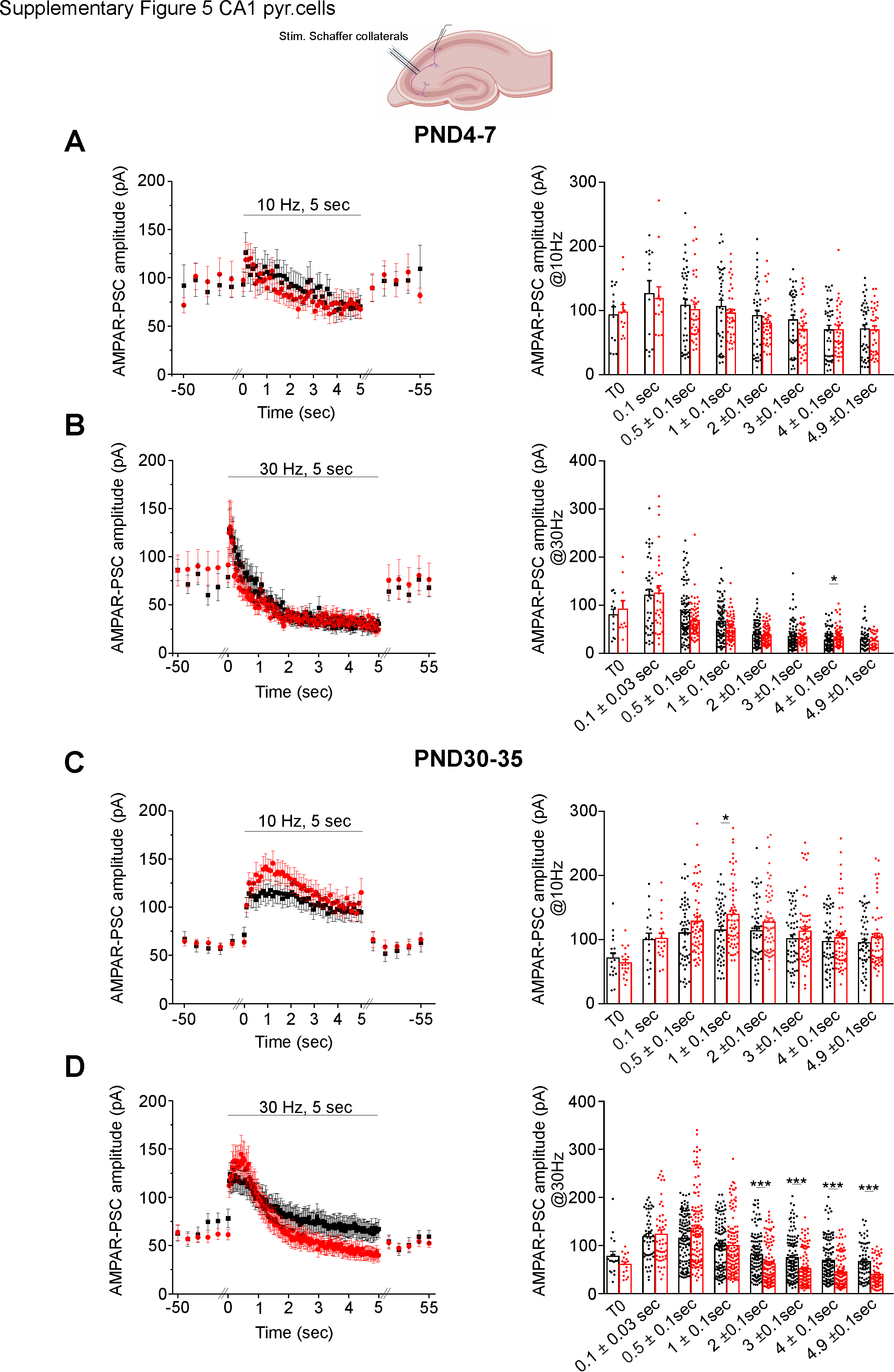

Supplement: SUPPLEMENTARY FIGURE 5 — Top: Schematic of the recorded region and experimental design (Biorender). (A–D) Absolute amplitudes of AMPAR-PSCs (not normalized data) recorded in CA1 pyramidal neurons evoked by electrical stimulation applied at 0.1, 10 and 30 Hz in the stratum radiatum from neonatal and juvenile WT (black) and Stxbp1+/− (red) mice. These data are at the origin of the graphs and plots showed in Figure 3 and Supplementary Figure 1. Statistics at t = 4 ± 0.1 sec (B): Mann–Whitney’s test p = 0.034*. Statistics at t = 2 ± 0.1 sec (D): Mann–Whitney’s test p = 0.0006***. Statistics at t = 3 ± 0.1 sec: Mann–Whitney’s test p < 0.0001***, Statistics at t = 3 ± 0.1 sec: Mann–Whitney’s test p < 0.0001***. Statistics at t = 4 ± 0.1 sec: Mann–Whitney’s test p < 0.0001***. Statistics at t = 4.9 ± 0.1 sec: Mann–Whitney’s test p < 0.0001***. [file Image_5.tif]

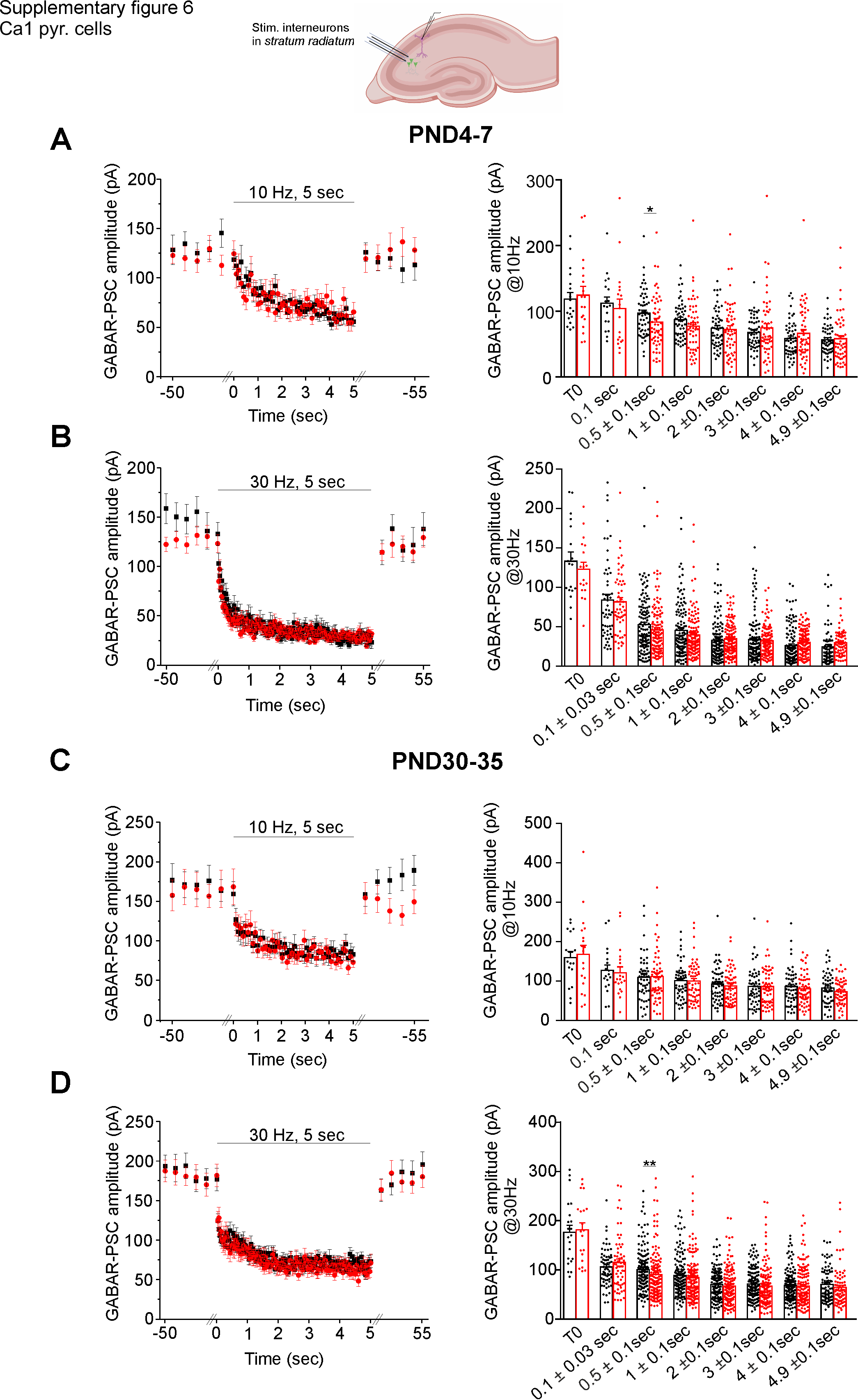

Supplement: SUPPLEMENTARY FIGURE 6 — Top: Schematic of the recorded region and experimental design (Biorender). (A–D) Absolute amplitudes of GABAR-PSCs (not normalized data) recorded in CA1 pyramidal neurons evoked by electrical stimulation applied at 0.1, 10 and 30 Hz in the stratum radiatum from neonatal and juvenile WT and Stxbp1+/− mice. These data are at the origin of the graphs and plots showed in Figure 4 and Supplementary Figure 2. Statistics at t = 0.5 ± 0.1 sec (A): Mann-Whitney’s test p = 0.035*. Statistics at t = 0.5 ± 0.1 sec (D): Mann–Whitney’s test p = 0.0053**. [file Image_6.tif]

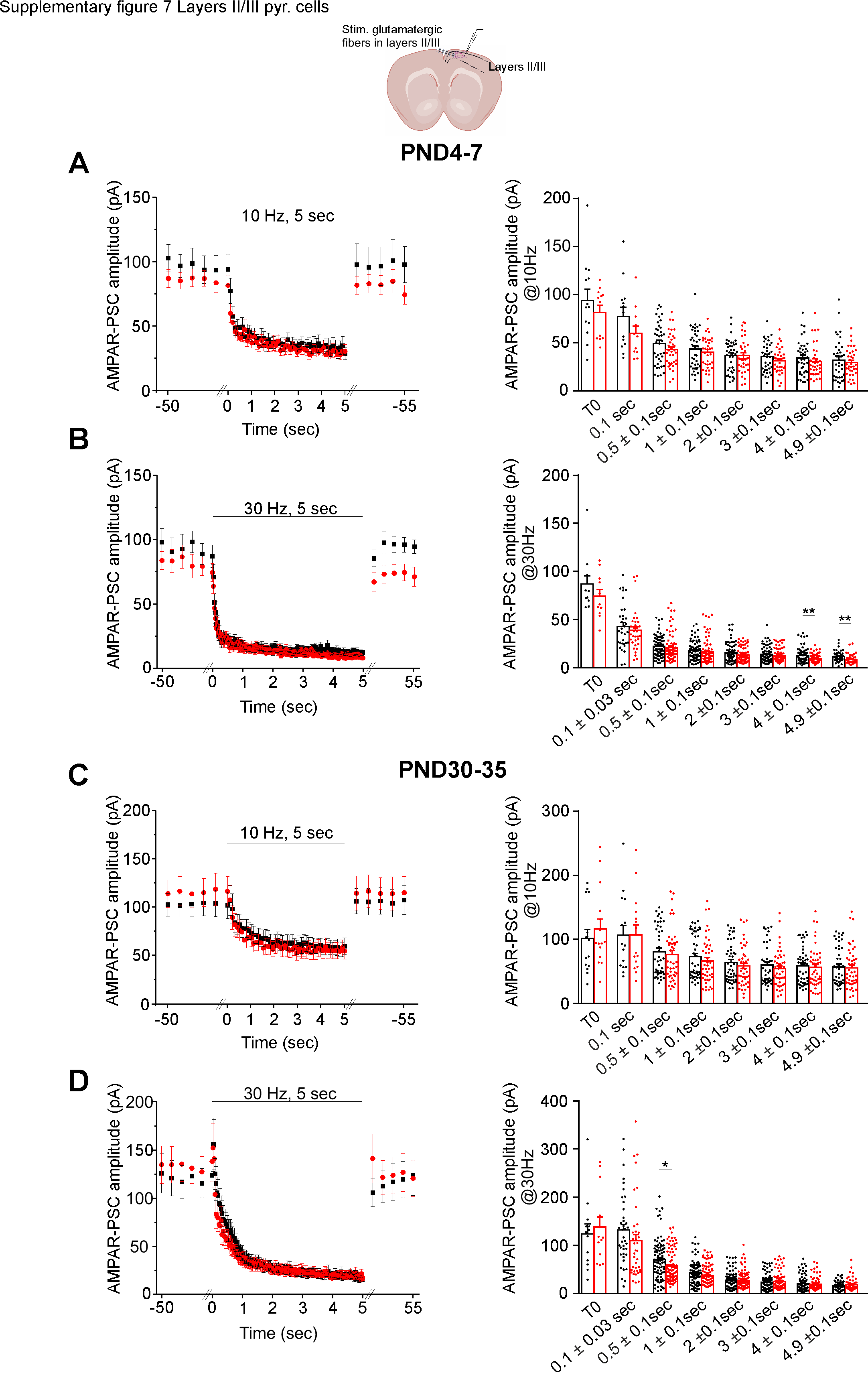

Supplement: SUPPLEMENTARY FIGURE 7 — Top: Schematic of the recorded region and experimental design (Biorender). (A–D) Absolute amplitudes of AMPAR-PSCs (not normalized data) recorded in layers II/III pyramidal neurons evoked by electrical stimulation applied at 0.1, 10 and 30 Hz in the layers II/III from neonatal and juvenile WT and Stxbp1+/− mice. These data are at the origin of the graphs and plots showed in Figure 5 and Supplementary Figure 3. Statistics at t = 4 ± 0.1 sec (B): Mann–Whitney’s test p < 0.0017**. Statistics at t = 4.9 ± 0.1 sec: Mann–Whitney’s test p < 0.002**. Statistics at t = 0.5 ± 0.1 sec (D): Mann–Whitney’s test p = 0.019*. [file Image_7.tif]

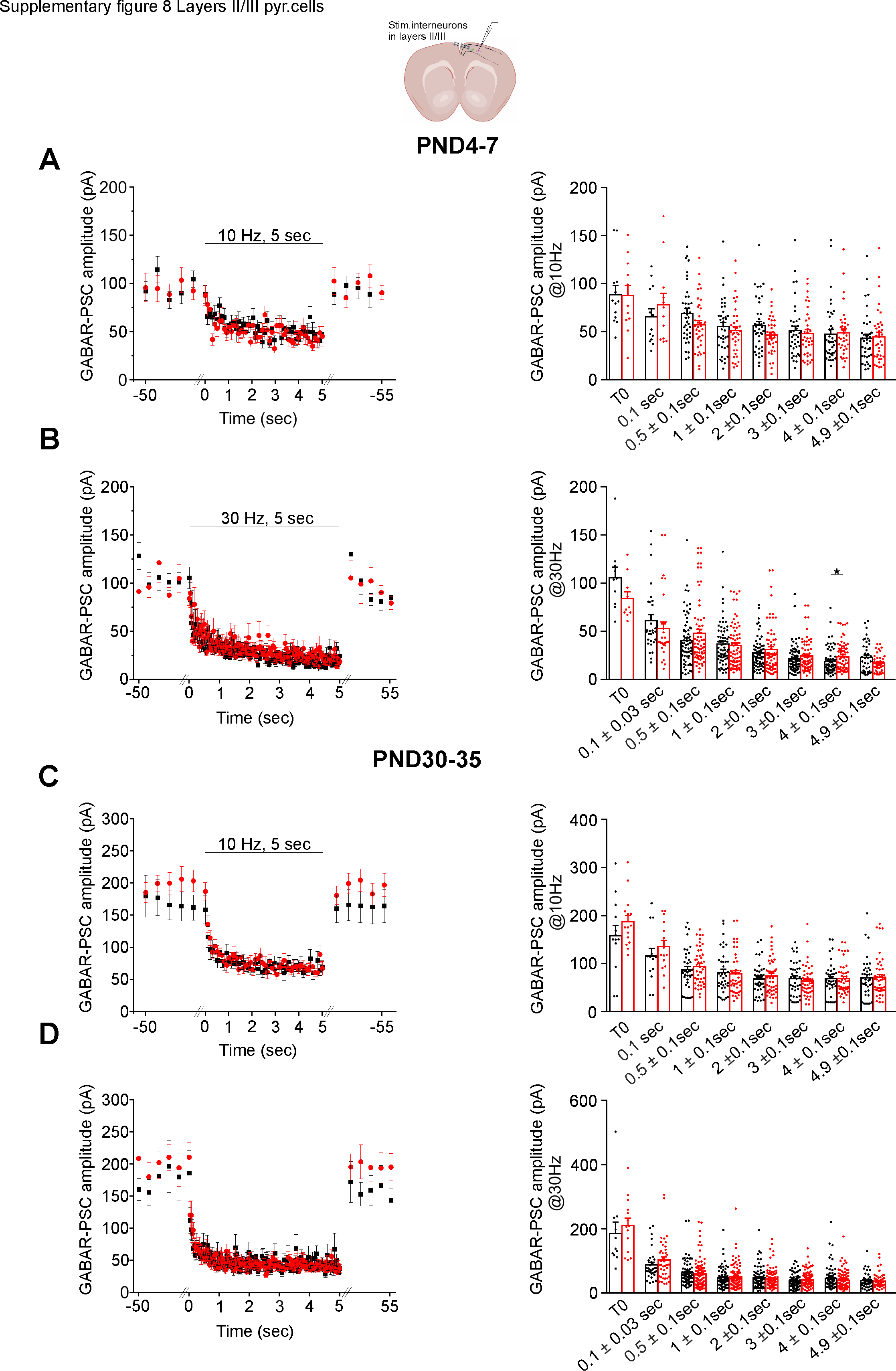

Supplement: SUPPLEMENTARY FIGURE 8 — Top: Schematic of the recorded region and experimental design (Biorender). (A–D) Absolute amplitudes of GABAR-PSCs (not normalized data) recorded in layers II/III pyramidal neurons evoked by electrical stimulation applied at 0.1, 10 and 30 Hz in the layers II/III from neonatal and juvenile WT and Stxbp1+/− mice. These data are at the origin of the graphs and plots showed in Figure 6 and Supplementary Figure 4. Statistics at t = 4 ± 0.1 sec (B): Mann–Whitney’s test p < 0.03*. [file Image_8.tif]

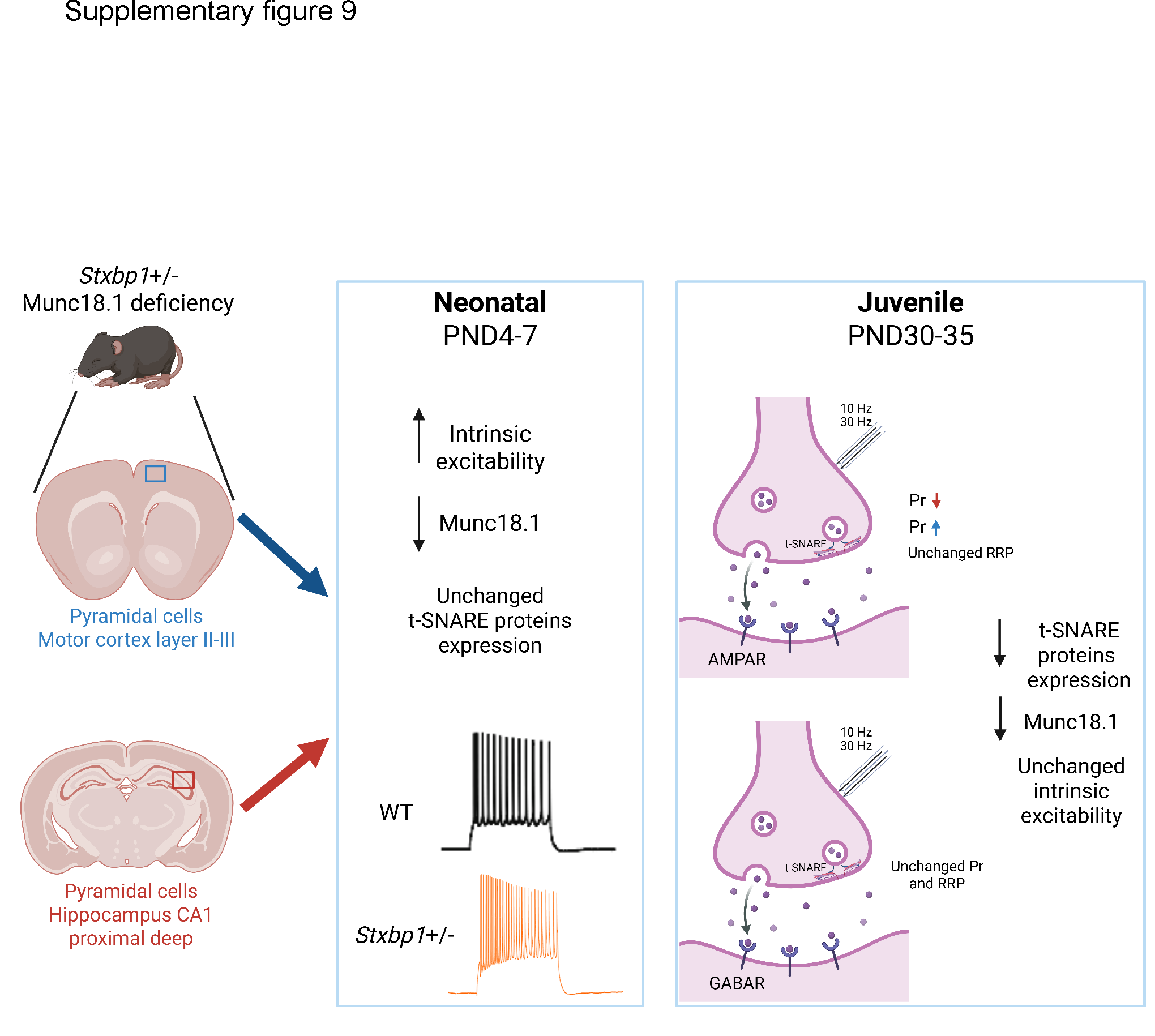

Supplement: SUPPLEMENTARY FIGURE 9 — Schematic summarizing findings (Biorender). [file Image_9.tif]
